# Evidence for a “Wattle and Daub” Model of the Cyst Wall of *Entamoeba*

**DOI:** 10.1371/journal.ppat.1000498

**Published:** 2009-07-03

**Authors:** Anirban Chatterjee, Sudip K. Ghosh, Ken Jang, Esther Bullitt, Landon Moore, Phillips W. Robbins, John Samuelson

**Affiliations:** 1 Department of Molecular and Cell Biology, Boston University Goldman School of Dental Medicine, Boston, Massachusetts, United States of America; 2 Department of Biotechnology, Indian Institute of Technology, Kharagpur, West Bengal, India; 3 Department of Biophysics and Physiology, Boston University School of Medicine, Boston, Massachusetts, United States of America; 4 Department of Genetics and Genomics, Boston University School of Medicine, Boston, Massachusetts, United States of America; University of California Los Angeles, United States of America

## Abstract

The cyst wall of *Entamoeba invadens* (Ei), a model for the human pathogen *Entamoeba histolytica*, is composed of fibrils of chitin and three chitin-binding lectins called Jacob, Jessie3, and chitinase. Here we show chitin, which was detected with wheat germ agglutinin, is made in secretory vesicles prior to its deposition on the surface of encysting Ei. Jacob lectins, which have tandemly arrayed chitin-binding domains (CBDs), and chitinase, which has an N-terminal CBD, were each made early during encystation. These results are consistent with their hypothesized roles in cross-linking chitin fibrils (Jacob lectins) and remodeling the cyst wall (chitinase). Jessie3 lectins likely form the mortar or daub of the cyst wall, because 1) Jessie lectins were made late during encystation; 2) the addition to Jessie lectins to the cyst wall correlated with a marked decrease in the permeability of cysts to nucleic acid stains (DAPI) and actin-binding heptapeptide (phalloidin); and 3) recombinant Jessie lectins, expressed as a maltose-binding proteins in the periplasm of *Escherichia coli*, caused transformed bacteria to agglutinate in suspension and form a hard pellet that did not dissociate after centrifugation. Jessie3 appeared as linear forms and rosettes by negative staining of secreted recombinant proteins. These findings provide evidence for a “wattle and daub” model of the *Entamoeba* cyst wall, where the wattle or sticks (chitin fibrils likely cross-linked by Jacob lectins) is constructed prior to the addition of the mortar or daub (Jessie3 lectins).

## Author Summary

Parasitic protists, which are spread by the fecal-oral route, have cyst walls that resist environmental insults (e.g. desiccation, stomach acids, bile, etc.). *Entamoeba histolytica*, the cause of amebic dysentery and liver abscess, is the only protist characterized to date that has chitin in its cyst wall. We have previously characterized *Entamoeba* chitin synthases, chitinases, and multivalent chitin-binding lectins called Jacob. Here we present evidence that the *Entamoeba* Jessie3 lectin contributes to the mortar or daub, which makes the cyst wall impenetrable to small molecules. First, the Jessie3 lectin was made after chitin and Jacob lectins had already been deposited onto the surface of encysting *Entamoeba*. Second, cysts became impenetrable to small molecules at the same time that Jessie3 was deposited into the wall. Third, recombinant Jessie3 lectins self-aggregated and caused transfected bacteria to agglutinate. These results suggest a “wattle and daub” model of the Ei cyst wall, where the wattle or sticks (chitin fibrils likely cross-linked by Jacob lectins) is constructed prior to the addition of the mortar or daub (Jessie3 lectins).

## Introduction

The infectious and diagnostic form of *Entamoeba histolytica* (Eh), the trophozoites of which cause amebic dysentery and liver abscess, is the cyst, which contains four nuclei surrounded by a chitin-containing wall [1]–[4]. *Entamoeba invadens* (Ei), which infects reptiles, is a model for encystation by Eh, because Ei readily form cysts when deprived of serum and other nutrients and salts in axenic culture without bacteria [5],[6]. In contrast, Eh encysts in an asynchronous manner in xenic cultures, which contain large numbers of bacteria and relatively few amebae [7]. In addition to chitin that is partially deacetylated to form chitosan, mass spectroscopy showed the Ei cyst wall contains three lectin families, members of which contain one or more Cys-rich chitin-binding domains (CBDs) that are unique to *Entamoeba* [8]–[11].

Seven Ei Jacob lectins (∼30% of cyst wall protein) are composed of three to seven CBDs, each of which contains six Cys and conserved aromatic residues [9],[11]. We hypothesize that Jacob lectins cross-link chitin fibrils, because all of the Jacob lectins have tandemly arranged CBDs, as do peritrophins, which are the major protein in chitin-based walls around the insect blood meal [12]. The CBDs of Jacob lectins are separated by Ser- and Thr-rich domains, which are modified by O-phosphodiester-linked glycans and contain conserved Cys-protease cleavage sites [11].

The Ei chitinase (∼20% of cyst wall protein) is composed of a single N-terminal CBD containing eight Cys residues, a low complexity spacer, and a C-terminal enzymatic domain that resembles those of yeast and fungi [10],[13]. The low complexity spacer of Eh chitinase contains heptapeptide repeats, which are polymorphic from isolate to isolate [14],[15]. The Ei genome also predicts two chitinases, which lack CBDs and are not present within the cyst wall [11],[16].

Two Jessie3 lectins, which are a focus of the present study, compose ∼50% of the protein mass of the Ei cyst wall [11]. Each Jessie3 lectin contains a single N-terminal 8-Cys CBD like that of Ei chitinase, a low complexity spacer, and a unique C-terminal domain of unknown function [10].

In encysting Ei, Jacob lectins and chitinase are present in large and small vesicles, respectively [9]. So-called “wall-less cysts” are formed in the presence of excess Gal, which inhibits binding of the plasma membrane Gal/GalNAc lectin to cyst wall glycoproteins including Jacob lectins and may also interfere with signaling in encysting Ei [9], [17]–[19]. Microarray analyses of Eh cysts formed in xenic cultures of recent isolates confirm that Jacob and Jessie3 lectins, as well as chitinases, are also encystation-specific proteins in the human pathogen Eh [7].

In the present study, we asked four questions concerning the assembly of the *Entamoeba* cyst wall. First, what is the order of addition of the various components of the cyst wall (chitin, chitinase, Jacob lectins, and Jessie lectins)? Second, are the lectins synthesized in the same or in different secretory vesicles? Are chitin fibrils made at the plasma membrane, as is the case in fungi [20],[21]? Third, is the unique C-terminal domain of the Jessie3 lectins an enzyme (e.g. chitinase or chitin deacetylase)? Alternatively, does the unique C-terminal domain of the Jessie3 lectin contribute to protein self-aggregation to form the mortar or daub in the cyst wall? Fourth, how do we synthesize the answers to these questions into a more complete model of the *Entamoeba* cyst wall?

## Results/Discussion

Previously we used confocal microscopy to show the Jacob lectin is present in large vesicles in encysting Ei, which are distinct from the small vesicles that contain chitinase [9]. Here we used 3D high-resolution microscopy to observe encysting Ei, which were fixed, permeabilized with non-ionic detergent, and then directly labeled with antibodies to Jessie3 lectins, Jacob lectins, and chitinase [9],[22]. In addition, the plant lectin wheat germ agglutinin, which binds to chito-oligosaccharides, was used to detect chitin fibrils. Because Ei trophozoites encyst in an asynchronous fashion, all time points are approximate in descriptions of the encysting process. In contrast, the order of appearance of the major cyst wall components is not approximate, as it was determined with double or triple labels for chitinase, Jacob, Jessie3, and/or chitin. Descriptions of various lectins in “small” or “large” vesicles are just that and do not imply an interpretation as to the nature or function of these vesicles (e.g. ER, Golgi, secretory vesicles, lysosomes, or chitosomes). Indeed the size of the vesicles appeared to simply reflect the relative abundance of the proteins present within them.

### Chitin is made in secretory vesicles prior to its deposition into the cyst wall

**Figure 1:**
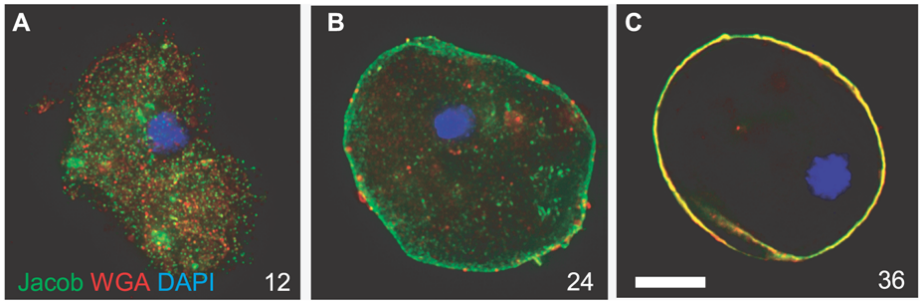
Three-dimensional high-resolution fluorescence microscopy shows that Jacob lectins and chitin are each synthesized in discrete vesicles and deposited onto the parasite surface early during Ei encystation. Parasites were fixed and permeabilized with nonionic detergent prior to labeling with antibodies to Jacob lectins (green), wheat germ agglutinin (red) that binds chitin, and DAPI (blue) that stains nuclei. Because encystation is not well-synchronized, in each case multiple labels were used on the same set of organisms in order to determine the order of events during encystation. Jacob lectins and chitin, which are absent in trophozoites (not shown), are each present in separate vesicles after 12 hrs of encystation. At 24 hrs there is substantially more Jacob lectin than chitin on the protist surface, while both Jacob and chitin are present in the wall of Ei encysting for 36 hrs. Bar is 10 microns. (A) is a composite of multiple optical sections, while (B and C) are each a single optical section.

Chitin and the chitin-binding lectins present in the cyst wall (Jacob, Jessie, and chitinase) were all absent from Ei trophozoites (data not shown). Early during encystation (for example, in the organism encysting for 12 hrs in Fig. 1A), chitin and Jacob lectins were present in separate vesicles. The presence of chitin in secretory vesicles of Ei, which was confirmed by examining serial optical sections of stained organisms, has also been observed using the chitin-binding stain calcoflour [23]. In contrast, chitin synthases of *Saccharomyces* are stored in chitosomes, but chitin is synthesized only at the plasma membrane [20],[21]. By 24 and 36 hrs, both chitin and Jacob lectin were rapidly accumulating in the Ei cyst wall, which became oval-shaped as in mature cysts (Figs. 1B and 1C). In contrast, there was little or no Jessie3 lectin in the Ei cyst wall at 48 hrs.

### Jacob lectins and chitinase are synthesized early during encystation

**Figure 2:**
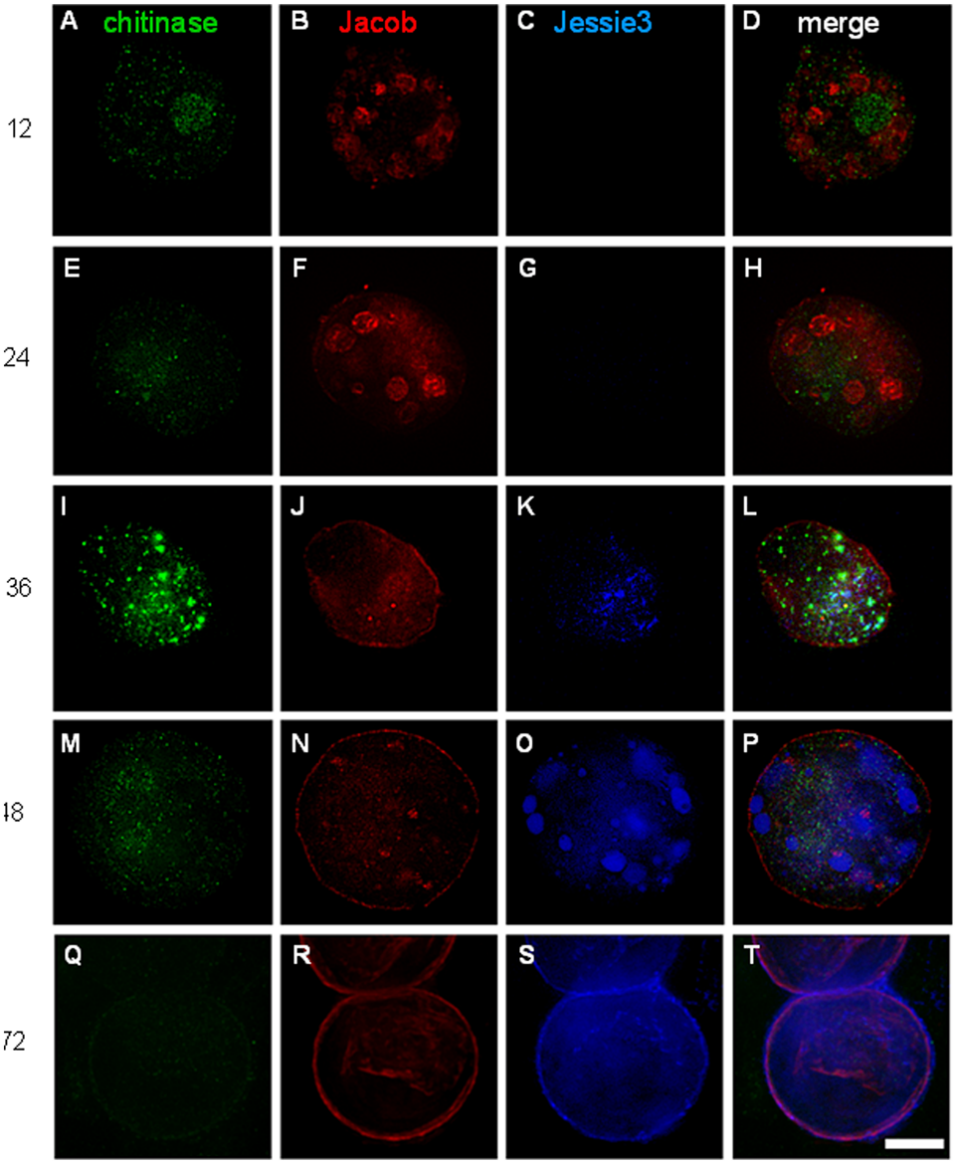
Three-dimensional high-resolution fluorescence microscopy shows Jacob lectins and chitinase are expressed early during encystation of Ei in vitro, while Jessie lectins are expressed late during encystation. Ei were allowed to encyst for 12 hrs (A to D), 24 hrs (E to H), 36 hrs (I to L), 48 hrs (M to P), or 72 hrs (Q to T) before they were fixed, permeabilized with non-ionic detergent, and then directly labeled with red anti-Jacob antibodies, green anti-chitinase antibodies, and blue anti-Jessie3 antibodies. At each time point, the same organism is shown with the three labels, and the merged three-color images are shown in the right hand-column. Not shown are control Ei trophozoites, which do not bind antibodies to Jacob, chitinase, or Jessie3. Also not shown are negative controls with non-immune rabbit antibodies, which did not bind to encysting organisms. Jacob lectins form large vesicles early during encystation; Jacob is the first lectin to appear on the surface of encysting Ei; and Jacob is a major component of the mature cyst wall. Chitinase appears next and is present in large vesicles that rarely overlap with those of Jacob lectins. In contrast to Jacob, most of the chitinase does not remain on the surface of mature cysts. Vesicles containing Jessie3 lectins appear late, and for the most part Jessie3 lectins are targeted to the cyst wall. Bar is 10 microns. Each image is a composite of multiple optical sections.

At early time points of encystation (12 to 24 hrs), Jacob lectins were present in large vesicles and began to appear on the surface of encysting Ei at the same time that chitin was present (Figs. 1A, 1B, and 2A to 2H). At the approximate midpoint of encystation (36 to 48 hrs), Jacob lectins were the major protein component of the Ei cyst wall (Figs. 1C and 2I to 2P). Later when cysts walls were completed at 72 hrs, both Jacob lectins and Jessie lectins were major components (Figs. 2Q to 2T). These kinetics are consistent with the idea that Jacob lectins, which have multiple tandemly arranged CBDs like those of peritrophins, are involved in cross-linking chitin fibrils [9]–[12].

While chitinase was also made early during encystation, chitinase was present in vesicles that were distinct from those of Jacob lectins (Figs. 2A to 2L). For the most part, chitinase was difficult to detect in Ei cyst walls and was nearly absent in secretory vesicles of fully developed cysts (Figs. 2M to 2T). These morphological results are slightly in conflict with mass spectroscopy of purified cyst walls, which suggested chitinase is relatively abundant [11]. These results are consistent with previous inhibitor studies, which show Ei chitinases are involved in remodeling the cyst wall as it is formed, as described for chitinases of fungi [12],[24],[25]. These results suggest the possibility that chitinases are not involved in excystation. Consistent with this idea, we were unable to inhibit excystation with the chitinase inhibitor allosamadin (data not shown). As shown below, it is also possible that allosamidin did not penetrate the wall of fully formed cysts.

### The late appearance of Jessie3 lectins in cyst walls correlates with a reduced permeability of Ei cysts to small molecules

**Figure 3:**
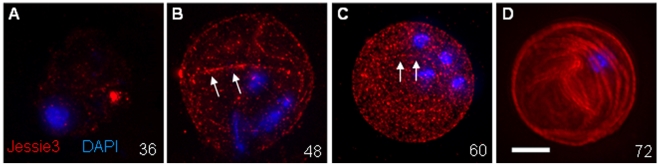
Three-dimensional high-resolution fluorescence microscopy shows that Jessie lectins (red) arrive late but eventually cover the surface of cysts. Unlike encysting Ei in Figs. 1, 2, and 4, protists here were labeled with antibodies to Jessie3 prior to fixation, so that only the surfaces of the encysting parasites are labeled. While there is very little Jessie3 lectin on the parasite surface after 36 hrs (A), Jessie3 appears in increasing number of punctate spots, sometimes in linear arrays (arrows), in the cyst wall at 48 hrs (B) and 60 hrs (C). In contrast, at 72 hrs (D), Jessie3 has a swirling appearance in the Ei cyst wall. After Jessie3 is fully incorporated into the wall (D), the cyst becomes impermeable to DAPI, so that nuclei are no longer visible or are weakly visible. Bar is 10 microns. Each image is the composite of multiple optical sections.

The synthesis of Jessie3 lectins was delayed in encysting Ei, so at 36 to 48 hrs, when Jacob lectins and chitin were already in the Ei cyst wall, Jessie3 lectins were restricted for the most part to secretory vesicles (Figs. 1A to 1C and 2A to 2P). In order to better visualize Jessie3 as it was added to the cyst wall, anti-Jessie3 antibodies were incubated with encysting Ei prior to fixation or permeabilization. These experiments confirmed the near absence of Jessie3 on the surface of encysting Ei at 36 hrs (Fig. 3A). At 48 and 60 hrs, Jessie3 lectins appeared as punctate spots on cyst wall, sometimes in linear arrays, which increased in density with time (Figs. 3B and 3C). At 72 hrs when encystation was complete and both Jacob and Jessie3 lectins stained the cyst wall (Figs. 2Q to 2T), Jessie3 lectins changed from a punctate to a continuous pattern and had a whorl-like appearance on the surface of cysts. By negative staining, Jessie3 formed linear arrays on the surface of cysts (data not shown). Because chitin fibrils were not well-visualized by these methods, we assume but cannot prove that Jessie3 is binding to individual chitin fibrils.

**Figure 4:**
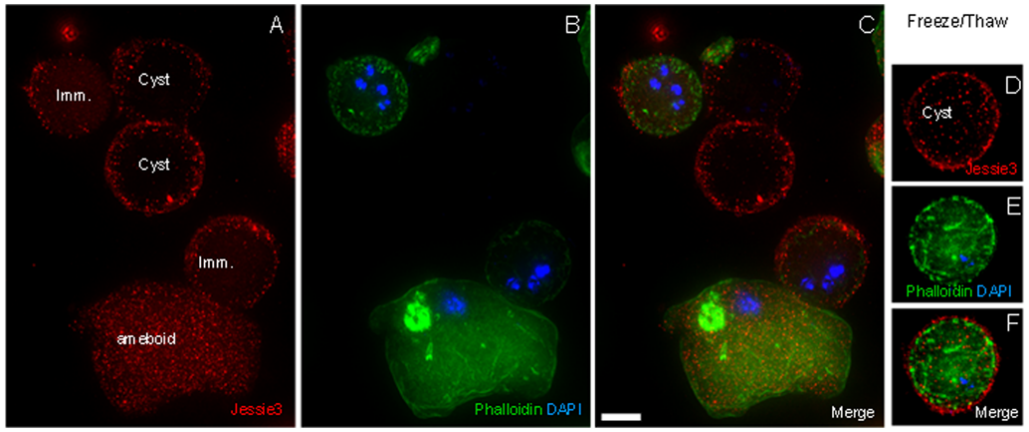
Three-dimensional high-resolution fluorescence microscopy shows that coincident with the addition of Jessie lectins (red), cyst walls become impermeable to DAPI (blue) and phalloidin (green). (A to C) After 72 hrs encystation, some Ei are still ameboid in appearance, some have relatively little Jessie3 lectin in the wall, and some protists have abundant Jessie3. DAPI and phalloidin stain well the immature cysts (Imm.) but fail to penetrate mature cysts. (D to F) In contrast, DAPI and phalloidin penetrate all cysts that have been frozen and thawed prior to staining with these reagents. Bar is 10 microns. Each image is a single optical section.

Ameboid forms and early cysts, which contain small amounts of Jessie3 lectins in their walls, label with DAPI and phalloidin, which bind to nuclei and actin fibrils, respectively, after fixation and permeabilization with non-ionic detergent (Figs. 4A to 4C). In contrast, late cysts, which contain abundant Jessie3 and Jacob in the cyst wall, do not label with DAPI or phalloidin until the cysts have been frozen and thawed to introduce an ice artifact that allows the dyes to penetrate the cyst wall (Fig. 4D to 4F). Examined from another point of view, ameboid forms are lysed by 0.1% SDS or sarcosyl, while the spherical cyst-like forms are resistant to ionic detergents (data not shown). However, treatment of early cysts with ionic detergents greatly reduces their capacity to excyst, while late cysts are more able to excyst after treatment with detergent.

Because both Jacob and Jessie3 lectins are each abundant in the walls of mature cysts, we cannot determine which abundant protein is responsible for the impenetrability of Ei cysts to small molecules. However, experiments with recombinant Jacob and Jessie3 lectins (next section) show that the latter self-aggregates, consistent with a possible role as the mortar or daub that seals the wall.

### Maltose-binding protein-EhJessie3 fusion-proteins self-aggregate and cause transformed bacteria to agglutinate

**Figure 5:**
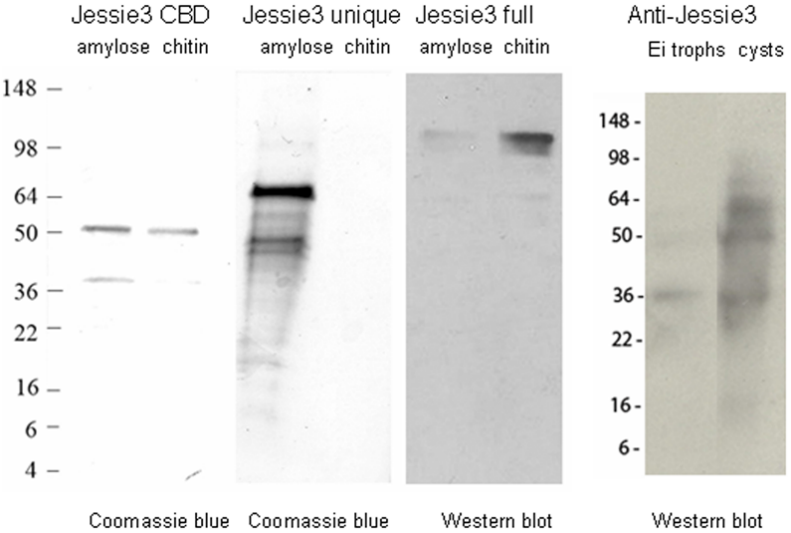
Chitin-binding is associated with the N-terminal Cys-rich domain of Jessie3. Left panel: Coomassie blue stained SDS-PAGE shows an MBP-fusion-protein containing the CBD of Jessie3, which was purified with an amylose resin. A lower mol wt band is likely MBP alone, as only the MBP-Jessie3 CBD fusion-protein binds to chitin (right lane). Left center panel: A Coomassie-stained MBP-fusion-protein containing the C-terminal unique domain of Jessie3, which was purified with the amylose resin (left lane), fails to bind to chitin (right lane). Right center panel: A Western blot with polyclonal rabbit antibodies to Jessie3 shows that an MBP-fusion-protein containing full-length Jessie3 self-aggregates, so that it is difficult to purify on the amylose resin (left lane). However, the MBP-full-length Jessie3 fusion-protein binds to chitin (right lane). Right panel: Polyclonal rabbit anti-Jessie3 antibodies bind weakly to trophozoites of Ei (left lane) but bind strongly to an ∼60-kDa protein in encysting Ei (the expected size of Jessie3) (right lane). Lower molecular weight bands may reflect a cleavage product between the N-terminal CBD and the C-terminal unique domain, as we have previously shown that Jacob lectins of encysting Ei are often cleaved between CBDs [9]. There was no binding of a control non-immune sera to trophozoite or cyst proteins (not shown).

To better understand the domain structure of the Jessie3 lectin, we expressed full-length Eh Jessie3, the putative N-terminal CBD domain, and the unique C-terminal domain as maltose-binding protein (MBP)-fusion proteins in the periplasm of *Escherichia coli* [26]. The MBP-fusions with the N-terminal CBD or the full-length Jessie3 each bound chitin beads (Fig. 5), while the MBP-fusion with the unique C-terminal domain of Jessie3 did not bind chitin. These results are consistent with our previous demonstration that the N-terminal domain of the Eh Jessie3 lectin, when expressed as an epitope-tagged protein in transfected Eh, is sufficient for chitin-binding [10].

Neither the full-length Jessie3 lectin nor the Jessie3 unique C-terminal domain had chitinase or chitin deacetylase activity, using assays that had previously demonstrated such activity from lysed Eh or recombinant Eh enzymes [8],[13]. Although the N-terminal CBD of full-length Jessie3 has the expected lectin activity, we cannot rule out the possibilities that 1) the unique C-terminal domain is not properly folded and so lacks its usual enzymatic activity or 2) the unique C-terminal domain contains an enzymatic activity not tested here.

**Figure 6:**
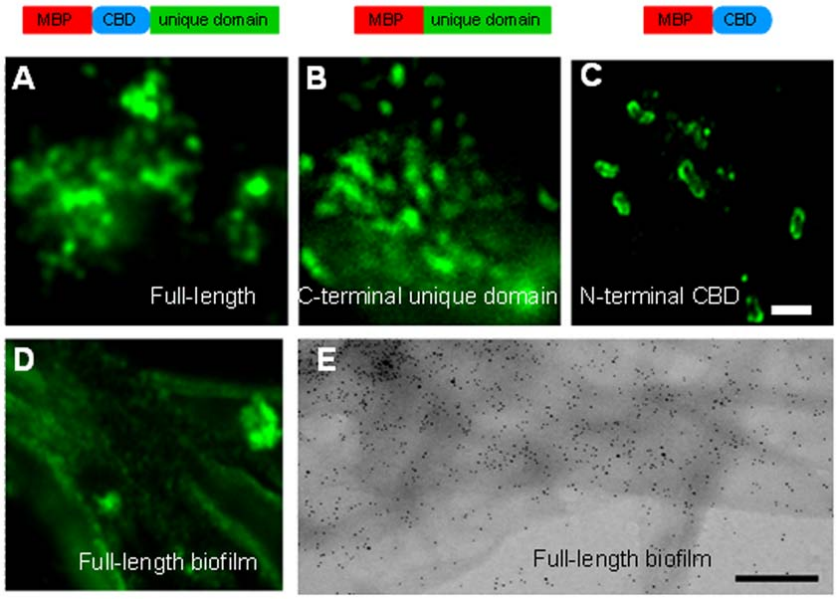
Recombinant Jessie 3 lectins self-aggregate. Fluorescence microscopy (A to D) and negative staining (E) of bacteria transformed with MBP-fusion-proteins expressing full-length Jessie3 (green in A) or the unique C-terminal unique domain of Jessie3 (green in B) agglutinate to form large clumps of bacteria. In addition, biofilms containing MBP-full length Jessie3 are formed (green in D and immunostained with gold in E). Antibodies to the unique C-terminal domain of Jessie 3 are conjugated to Alexafluor in (A, B, and D) or are detected with immunogold in (E). Antibodies to the N-terminal CBD of Jessie3 (green in C) show that MBP-fusion-proteins containing this domain do not self-aggregate on a macro-scale and do not agglutinate bacteria. Bar (A to D) is 2 microns. Bar (E) is 500 nm.

However, an alternative function for the unique C-terminal domain of Jessie3 was suggested by the finding that transformed bacteria expressing full-length Eh Jessie3 agglutinate in solution. After centrifugation in a microfuge at high speed, these bacteria aggregated into a solid pellet, which did not dissociate with vortexing or pipetting. Transformed bacteria expressing MBP-fusions containing only the unique C-terminal domain of Jessie3 also agglutinated in solution and aggregated into an insoluble pellet after centrifugation. Anti-Jessie3 antibodies and fluorescence microscopy showed agglutinated *E. coli* secrete large amounts of the MBP-Jessie3 fusion-proteins, which accumulated in sheets or biofilms on the surface of the slide or cover slip (Figs. 6A, 6B, and 6D). Negative staining showed secreted MBP-full-length Jessie3 fusion-proteins also formed large planar aggregates that labeled with gold-conjugated secondary antibodies after primary antibody labeling of the Jessie3 lectin (Fig. 6E). In contrast, bacteria expressing MBP only, MBP fused to the N-terminal CBD of Jessie3, or MBP fused to multiple CBDs of the Jacob lectin did not agglutinate bacteria in solution, did not form a solid pellet after centrifugation, and failed to make biofilms on slide surfaces (Fig. 6C and data not shown). These results strongly suggest that the unique C-terminal domain of Eh Jessie3 is an important contributor to bacterial agglutination in solution and insoluble pellet-formation after centrifugation.

**Figure 7:**
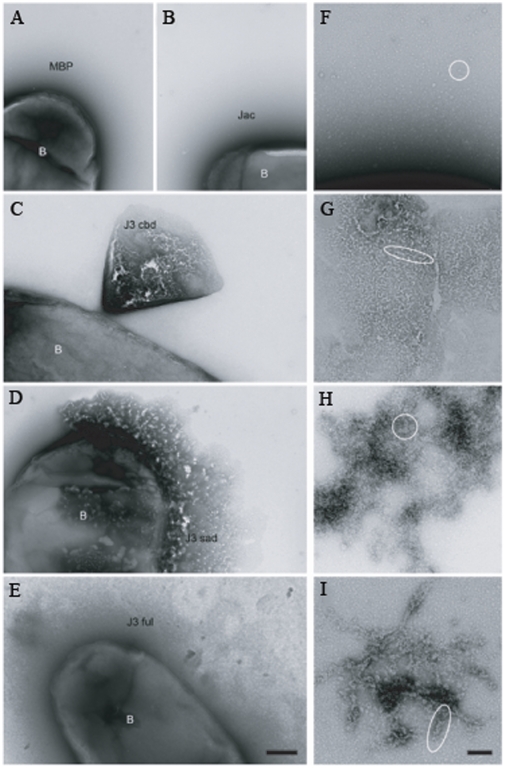
Negative stains of bacteria transformed with MBP constructs targeted to the periplasm suggest the unique C-terminal domain of Jessie3 is an important contributor to self-aggregation. Bacteria (B) expressing MBP alone (low magnification in A and high magnification in F) or an MBP-Jacob2 fusion-protein (jac in low magnification in B) release dense quantities of proteins, which do not self-aggregate. In contrast, bacteria expressing an MBP-Jessie3 CBD fusion-protein (J3 CBD in low magnification in C and high magnification in G) release proteins in conical shaped eruptions from the periplasm, and the MBP-Jessie3 CBD fusion-proteins form short, thin linear arrays. Bacteria expressing MBP-Jessie3 unique C-terminal domain fusion-proteins (J3 sad (self-adhering domain) in low magnification in D and high magnification in H) or MBP-full-length Jessie3 (J3 ful in low magnification in E and high magnification in I) release dense aggregates of proteins, and sheets of these MBP-Jessie3 fusion-proteins contain branched aggregates of multiple-stranded linear forms that are thicker than those formed by Jessie3 CBD. Bar (A to E) is 250 nm. Bar (F to I) is 100 nm. Circles and ovals are added to figures to highlight structures of secreted proteins.

We used negative staining to get a better view of the aggregated MBP-Jessie3 fusions. The full-length Jessie3 and the unique C-terminal domain of Jessie3 formed dense aggregates on the surface of transformed *E. coli* and often disrupted the outer bacterial membrane (Figs. 7D and 7E). While these aggregates did not form a higher order crystal structure, MBP-full-length Jessie3 fusion-proteins made linear structures that aggregated into larger branched structures (Fig. 7I). Similar linear and branched structures were formed by MBP-Jessie3 unique C-terminal domain fusion-proteins, although the branches were not so long (Fig. 7H). In contrast, control MBP alone and MBP-Jacob2 lectin fusion-proteins stayed in solution and did not self-aggregate (Figs. 7A, 7B, and 7F). Interestingly, MBP fusion-proteins containing the N-terminal CBD of Jessie3 often formed cone-shaped aggregates, as they were released from the periplasm of *E. coli* (Fig. 7C). These MBP-Jessie3 CBD fusion-proteins formed smaller self-aggregates and thinner linear forms than the MBP-full length Jessie3 fusion-proteins (Fig. 7G).

We conclude that the C-terminal unique domain of Jessie3 appears to be responsible for most protein self-aggregation and integrate these findings into a revised model of the Ei cyst wall (next section).

### A “wattle” (chitin fibrils and Jacob lectins) and “daub” (Jessie3 lectins) model of the *Entamoeba* cyst wall

**Figure 8:**
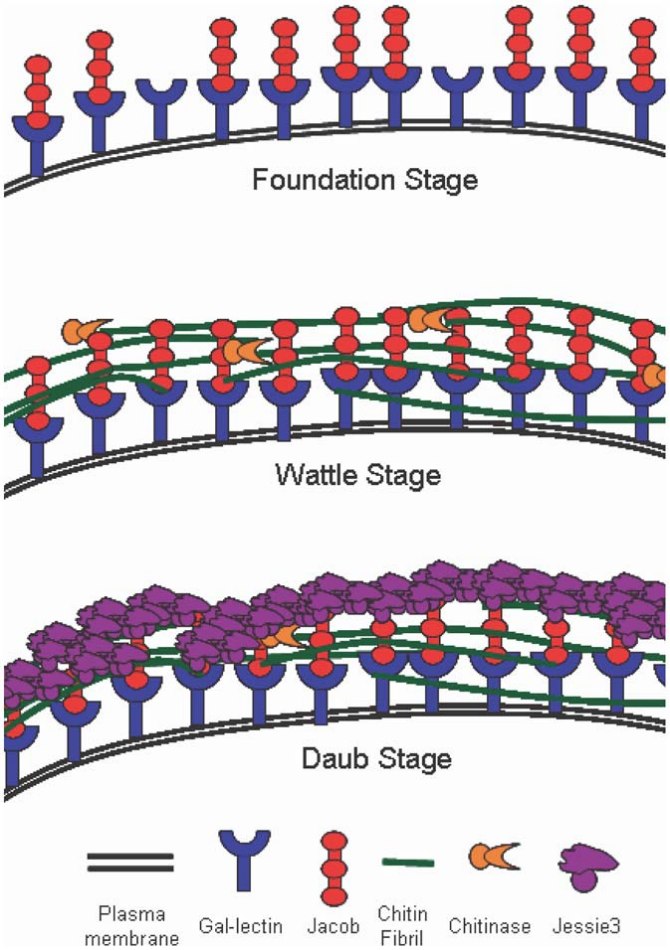
“Wattle and daub” model of the Ei cyst wall (a hypothesis). In the first “foundation” phase of encystation, Jacob lectins, which are themselves glycoproteins that contain Gal, are bound to the surface of encysting amebae by constitutively expressed plasma membrane Gal/GalNAc lectins [9],[16]. In the second “wattle” phase, Jacob lectins, which contain tandemly arranged CBDs, appear to cross-link chitin fibrils that are deposited on the surface of encysting amebae. In the third “daub” phase, the cyst wall is solidified and made impermeable to small molecules by the addition of the Jessie3 lectin, which has an N-terminal CBD that binds chitin and a unique C-terminal unique domain that appears to cause self-aggregation.

The results here and elsewhere suggest that the cyst wall of Ei may be made in three phases (Fig. 8). During the first “foundation” phase, Jacob lectins, which are encystation-specific glycoproteins that contain Gal, are bound to the surface of encysting amebae by Gal/GalNAc lectins that are constitutively expressed [9], [16]–[18]. This idea is supported by our previous demonstration that Ei form “wall-less cysts” in the presence of excess galactose and by demonstration that the Gal/GalNAc lectin binds Jacob lectins on Western blots [9]. In the second “wattle” phase, chitin is synthesized, secreted, and is bound the surface of encysting amebae. There chitin fibrils are likely cross-linked by Jacob lectins, which contain multiple tandemly arranged CBDs, although cross-linking has not been proven [9],[10]. During the third “daub” phase, the cyst wall is solidified and may be made impermeable to small molecules by the addition of the Jessie3 lectin, which has a single CBD that binds chitin fibrils and a unique C-terminal domain that appears to promote self-aggregation. As already mentioned, we cannot rule out a role for the Jacob lectin in the formation of the mortar or daub in the Ei cyst wall. We cannot rule out some other biochemical events (e.g. chemical cross-linking), which might affect cyst wall permeability.

Other biochemical events, which we have not assigned to particular stages in the model presented here include: 1) Jacob lectins are cleaved into smaller repeats by the action of proteases that recognize conserved sequences between CBDs [11],[27],[28], and 2) chitin is trimmed by chitinase and deacetylated by chitin deacetylase [8],[13]. Although the “wattle and daub” model is supported by numerous observations and experiments, we do not presently have the technology to knock out or knock down gene expression in Ei, which might more conclusively prove the essential role of each component of the Ei cyst wall.

The “wattle and daub” model for the *Entamoeba* cyst wall shows little resemblance to models of the walls of fungi, plants, or slime molds [29]–[34]. This is likely the case because the *Entamoeba* cyst wall, which appears to be homogenous rather than multi-layered, contains a single sugar homopolymer (chitin) and a small set of chitin-binding lectins (Jacob, Jessie, and chitinase). In contrast, the walls of fungi, plants, and slime molds have multiple layers, numerous sugar homopolymers (chitin, cellulose, glucans, and/or GalNAc polymers), and numerous enzymes, structural proteins, and large oligosaccharides (e.g. mannans). As noted above, *Entamoeba* appears to make chitin within secretory vesicles rather than at the plasma membrane, as described in fungi [20],[21],[23].

This model has been developed using encysting Ei and bacterially expressed Eh Jessie3 and Jacob lectins. It is likely that numerous aspects apply to Eh, the human pathogen that does not readily encyst in culture, for the following reasons. First, Eh has the same parts list of chitin synthase, chitinase, chitin deacetylase, Jacob and Jessie3 lectins, and chitinase as Ei [4], [8]–[11],[13],[16],[35]. Second, Eh cysts from clinical samples stain with antibodies to Jacob lectins and Jessie3 lectins (our unpublished data and [9]). Third, Eh encysting in xenic cultures (filled with bacteria) express numerous mRNAs for Jacob and Jessie lectins, and chitinase [7]. In human studies and in mouse models, antibodies to the Gal/GalNAc lectin appear to protect the host from amebic infection [3],[17],[36]. Whether antibodies to the Gal/GalNAc lectin or antibodies to cyst wall lectins (Jacobs, Jessie3, or chitinase) interfere with cyst wall formation during clinical infection is an important unanswered question.

## Materials and Methods

### Deconvolving microscopy of Jessie3, Jacob, chitinase, and chitin in encysting *Entamoeba*

Trophozoites of the IP-1 strain of Ei were grown at 25°C in axenic culture in TYI-SS medium. Ei encystation was induced by placing parasites for 12–72 h in low-glucose (LG) medium, which has reduced osmolarity, glucose, and serum levels with respect to TYI-SS medium [37]. To test for excystation, cysts were washed with sterile water to lyse trophozoites, and then cysts were placed in TYI-SS medium for 2 hrs at 37°C. In addition, cysts were treated with 0.1% SDS and then washed and excysted, as described above.

Ei trophozoites and cysts, which contain four nuclei and have a chitin wall at maturity, were incubated with antibodies or lectins after washing in phosphate-buffered saline (PBS) or after fixation for 10 min in 2% paraformaldehyde at 4°C in the presence of 0.1% Triton X-100 to permeabilize membranes [9].

Mono-specific polyclonal antibodies to Ei Jacob were made previously by immunizing rabbits with Jacob lectin purified from a two-dimensional protein gel of cyst walls [9]. Mono-specific rabbit antibodies were made previously to a multi-antigenic peptide containing chitinase repeats [22].

Here rabbits were immunized with either the N-terminal CBD of Eh Jessie3 or its unique C-terminal domain, each of which was expressed as a fusion-protein with the maltose-binding protein (MBP) of *Escherichia coli* at the N-terminus (see below) [26]. MBP-Jessie3 fusion-proteins were purified on amylose resins, checked by SDS-PAGE, and sent to Strategic Biosolutions for mono-specific polyclonal rabbit antibody production.

Prior to their use in microscopy, rabbit antibodies were purified using MBP-Jessie3 fusion-proteins chemically coupled to agarose. Western blots showed binding of the anti-Jessie3 antibody to an MBP-full-length Jessie3 fusion-protein and to trophozoites and cysts of Ei (Fig. 5). Rabbit antibodies were then directly labeled red, green, or blue with Alexafluor dyes, diluted to a concentration of 1 mg/ml, and incubated with trophozoites or cysts for 60 mins at room temperature in PBS+2% bovine serum albumin (BSA). Parasites were washed four times in PBS prior to staining of the walls, nuclei, or cytoskeleton.

Chitin in encysting Ei and in cyst walls was stained with Alexafluor-conjugated wheat germ agglutinin at a concentration of 20 µg per ml for 60 min at 4°C in PBS-BSA. Cyst nuclei were visualized with 4′,6-diamidino-2-phenylindole (DAPI), while actin filaments were visualized with 1 µg per ml fluorescein-isothiocyanate-conjugated phalloidin. To determine whether the Ei cyst wall causes a permeability barrier to small molecules, Ei cysts were stained as described above, or Ei cysts were fixed and then exposed to three cycles of freezing and thawing to create an ice artifact prior to labeling with DAPI (molecular weight (MW 277) or phalloidin (MW 3945 of pentamer).

Slides were examined by three-dimensional multiple wavelength fluorescence microscopy using an Olympus IX70 microscope equipped for Deltavision deconvolution (Applied Precision). This system employs restorative as well as deconvolution techniques to provide resolutions up to four times greater than conventional light microscopes and is used to study the ultrastructure of intracellular structures such as the kinetochore [38],[39]. Images were collected at 0.2 mm optical sections for the indicated wavelengths and were subsequently deconvolved using SoftWoRx (Applied Precision). Data were examined as either optical sections or as a projection of the entire stack.

### Negative staining of Ei cysts

Ei cysts were sonicated, and cyst walls were purified on sucrose gradients, as described [9]. Purified cyst walls were then washed and resuspended in Tris-EDTA (TE) buffer, pH 7.5, and applied to carbon-coated, copper grids. The sample on the grid was allowed to adsorb for 5 minutes, washed with TE, and blocked with 2% BSA solution in TE. Grids were exposed to rabbit anti-Jessie3 antibodies, which were diluted 1∶20 dilution in TE, for 1 hr at RT, and then grids were washed six times in TE for 10 min. Grids were incubated with a secondary goat-anti rabbit antibody conjugated to a 5 nm gold particle for 1 hr at RT, followed by 6 washes with TE. The grids were stained with 0.1% uranyl acetate for 15 sec and visualized on a Phillips CM-12 microscope at 120 KV, 25,000 to 60,000 times magnification. Images were recorded on Kodak 50–63 film and digitized on a Nikon 9000 scanner at 2000 dpi.

### Expression of Jessie3 lectins in transformed bacteria

The Eh Jessie3 gene was PCR amplified using three sets of primers to amplify the N-terminal CBD (Fwd: CGGGATCCTTAAATATCACATTTAGTCAACG and Rev: GGGAAGCTTTTACTGATTGACTTGGTCTTC), the unique C-terminal domain (Fwd: TCTAGACTTCCATTAGTACTAAAATTTGA and Rev: AAGCTTTTAAGAAGAACATAAATTAGATCTTC), and the full length Eh Jessie3 sequence (Fwd: GCTCTAGATTAAATATCACATTTAAGTCAACGAACC and Rev: GGATCCTTAGTGGTGGTGGTGGTGGTGTTTTGAATAATGTTCTTGTTTGT). The three Eh Jessie3 PCR products were cloned into pMAL-p2E vector (New England Biolabs (NEB), Beverley, MA), which makes an IPTG-inducible, periplasm-targetted fusion-protein with MBP at the N-terminus and Jessie3 at the C-terminus. Bl21-DE3 cells from Invitrogen were transformed with the pMAL-p2E-Jessie3 constructs, and recombinant proteins were induced with 0.1 mM IPTG. Fusion-proteins were extracted by sonication (5–10 pulses) of bacteria in phosphate-buffer (PB) and 4 mM PMSF on ice. Fusion-proteins were purified by 1) anion exchange chromatography through DEAE-Sepharose resin using 200 mM KCl in PB as an eluent and 2) amylose resin (NEB) using 100 mM maltose as an eluent. Enriched proteins were checked for purity on SDS-PAGE stained with Coomassie brilliant blue and by Western blot using horse-radish peroxidase- (HRP-) tagged anti-MBP antibody from NEB.

### Indirect immunoflourescence and negative staining of bacteria expressing Jessie3 lectin

*E. coli* Bl21-DE3 cells expressing Eh Jessie3 proteins were harvested and washed in PBS. For indirect immunofluorescence, bacteria were fixed with 2% paraformaldehyde in PBS on ice for 5 min. The fixed cells were incubated with polyclonal mono-specific rabbit antibody to MBP-Jessie3 CBD fusion-proteins at a dilution of 1∶100 for 1 hr at RT and washed three times in PBS. For secondary antibody, the cells were incubated with goat anti-rabbit antibody conjugated to Alexafluor for 1 hr at RT and washed three times with PBS.

For negative staining, unfixed *E. coli* cells expressing Jessie3 protein were resuspended in TE buffer, applied to grids, and incubated with anti-Jessie antibodies, as described for Ei cyst walls (above). Alternatively, *E. coli* and Jessie3 proteins secreted into the medium were visualized by negative staining in the absence of anti-Jacob antibodies.

### Methods for assaying for chitin-binding, chitinase, and chitin deacetylase activities

Chitin-binding assays for fusion-proteins containing MBP-full length Jessie3, MBP-Jessie3 unique C-terminal domain, MBP-Jessie3 N-terminal CBD, and MBP only (negative control) were performed using methods similar to those described in refs. 9 and 10. Briefly, recombinant proteins, which were purified from bacterial lysates on an amylose resin, were incubated with chitin beads (NEB), washed, and then eluted by boiling in SDS. Eluted proteins were separated on SDS-PAGE and identified by Coomassie blue staining (MBP-Jessie3, MBP-Jessie N-terminal CBD, and MBP only). Because of problems of self-aggregation, MBP-full- length Jessie3, which was never very abundant after release from the amylose resin, was detected with mono-specific polyclonal antibodies to Eh Jessie3.

Recombinant MBP-Jessie3 unique domain fusion-proteins were assayed for probable chitinase activity using the method to determine cellular chitin in yeast [40]. Briefly, 5 µg of purified Jessie3 protein was incubated with 10 µg of chitin or hexa-N-acetyl-glucosamine (GlcNAc_6_) in presence of 50 µl of Mcllavaine's buffer (18 mM citric acid, 64 mM dibasic sodium phosphate, pH 6) at −20°C for 10 min and subsequently incubated for 4 hrs at 37°C. Fifty µl of water was added to the sample and centrifuged at 12000×g to produce a clear supernatant. The release of GlcNAc was measured by the Morgan-Elson method of incubating the supernatant with 10 µl Borax (2.7 M sodium borate) at 99°C for 10 min, followed by incubation with 100 µl of p-dimethylaminobenzaldehyde (DMAB) for 20 min at 37°C. Resulting samples were measured by a colorimetric assay at 585 nm. Chitinase C (Sigma) was used as a positive control.

For chitin deacetylase assay, 5 µg of purified Jessie3 protein was incubated with 25 mM chitin or GlcNAc_6_ in 50 mM Tris, pH 8 and 1 mM cobalt chloride buffer at 50°C for 16 hrs [8]. The sample was separated on a thin layer chromatography (TLC) plate with an n-butanol∶ethanol∶water∶acetic acid (5∶4∶3∶1) buffer system and stained with diphenylamine or ninhydrin.
